# In vivo interrelationships between the gluteus minimus and hip joint capsule in the hip internal rotation position with flexion

**DOI:** 10.1186/s12891-024-07188-5

**Published:** 2024-01-23

**Authors:** Masahiro Tsutsumi, Akari Saiki, Isao Yamaguchi, Akimoto Nimura, Hajime Utsunomiya, Keiichi Akita, Shintarou Kudo

**Affiliations:** 1 Inclusive Medical Sciences Research Institute, Morinomiya University of Medical Sciences, 1-26-16 Nankokita, Suminoe-ku, Osaka city, 559-8611 Japan; 2 Department of Physical Therapy, Morinomiya University of Medical Sciences, Osaka, Japan; 3 Department of Clinical Anatomy, Graduate School of Medical and Dental Sciences, Tokyo Medical and Dental University, Tokyo, Japan; 4 Department of Radiological Science, Faculty of Health Science, Morinomiya University of Medical Sciences, Osaka, Japan; 5 Department of Functional Joint Anatomy, Graduate School of Medical and Dental Sciences, Tokyo Medical and Dental University, Tokyo, Japan; 6 Tokyo Sports & Orthopaedic Clinic, Tokyo, Japan

**Keywords:** Anterior hip pain, Gluteus minimus, Hip internal rotation, Joint capsule

## Abstract

**Background:**

The flexion adduction internal rotation (FADIR) test is performed by the combined motions of hip flexion (with knee flexion), adduction, and internal rotation, and can often reproduce anterior hip pain consistent with an individual’s presenting pain. Since it has high sensitivity for intraarticular pathology diagnosis but low specificity, understanding the extraarticular pathology that can induce anterior hip pain in the FADIR test may also be essential. This study hypothesized that the interrelationships between the joint capsule and gluteus minimus differ in individuals with and without FADIR-positive pain and aimed to elucidate the in vivo interrelationships at hip internal rotation in 90°-flexion, which is also often restricted in individuals with FADIR-positive pain.

**Methods:**

Ten hips were included in the FADIR-positive group, and ten hips without hip pain in the FADIR test were included in a control group. Based on the ultrasound images at the four hip rotation conditions (20° and 10° external rotations, 0° external/internal rotation, and 10° internal rotation), orientation measurements of the gluteus minimus (muscle belly portion) and joint capsule were performed and quantitatively compared between the FADIR-positive and control groups. Additionally, 3 hips of 3 participants were randomly selected from each of the control and FADIR-positive groups for magnetic resonance imaging analysis.

**Results:**

At 0°-external/internal and 10°-internal rotation, on ultrasound images, fibers of the gluteus minimus and joint capsule in the FADIR-positive group were significantly more oriented in the same direction than those in the control group. Magnetic resonance imaging showed that the loose connective tissue between the gluteus minimus and joint capsule was prominent at 10°-internal rotation in the control group, although this was not apparent in the FADIR-positive group.

**Conclusions:**

At hip internal rotation in 90° flexion, the muscular belly portion of the gluteus minimus and joint capsule were oriented in the same direction to a greater extent in the FADIR-positive group than in the control group owing to a morphological change in the loose connective tissue between them. The pathological changes in the loose connective tissue may inhibit smooth movement of the gluteus minimus relative to the joint capsule in individuals with FADIR-positive pain.

**Supplementary Information:**

The online version contains supplementary material available at 10.1186/s12891-024-07188-5.

## Introduction

Hip pain pathology is generally divided into two broad categories: intraarticular and extraarticular pathologies. The flexion adduction internal rotation (FADIR) test is a useful physical examination for assessing intraarticular pathologies, such as femoroacetabular impingement syndrome due to hip bone deformities and associated acetabular labrum pathology [1–4]. Combined motions of hip flexion (with knee flexion), adduction, and internal rotation can often reproduce anterior hip pain consistent with an individual’s presenting pain complaint [1–4]. However, the FADIR test has low specificity for the diagnosis of femoroacetabular impingement syndrome and acetabular labral pathology, regardless of its high sensitivity [5, 6]. Thus, it is essential to understand extraarticular pathologies that can induce anterior hip pain on the FADIR test.

Individuals with anterior hip pain detected by the FADIR test often show limited hip internal rotation with the hip flexed to 90° [7–10]. In general, hip internal rotation restriction factors are considered to comprise hip posterior structures, such as the external rotator muscles and posterior joint capsule (ischiofemoral ligament) [11–13]. Consistent with this general theory, the cause of limited hip internal rotation in individuals with anterior hip pain is also interpreted as involving posterior structures; accordingly, there is a discrepancy between the pain location and causal site of limited hip internal rotation.

According to recent studies, pathological changes in the fat pad and loose connective tissue on the anterior inferior iliac spine are closely related to the development of anterior hip pain [14]. Additionally, interventions for these pathologies (e.g. surgery or ultrasound-guided injections) are reported as effective for not only anterior hip pain, but also hip movement, based on patient-reported outcomes [14, 15]. Therefore, the hypothesis that an anterior hip extraarticular structure, namely this loose connective tissue, may be related to both anterior pain on the FADIR test and the range of motion in hip internal rotation should be considered.

This loose connective tissue on the anterior inferior iliac spine is distributed distally in the region surrounded by the joint capsule and pericapsular muscles (e.g. the proximal rectus femoris, iliopsoas, and gluteus minimus), and shows postural differences according to hip position [16, 17]. Additionally, this loose connective tissue has been visualized in vivo using ultrasound and magnetic resonance (MR) imaging analysis [16, 17]. Therefore, interrelationships via the loose connective tissue between the joint capsule and pericapsular muscles, especially the gluteus minimus, which is a hip rotator, can be analyzed by ultrasound and MR imaging, and may differ between individuals with and without FADIR-positive pain.

This study aimed to elucidate the in vivo interrelationships between the joint capsule and gluteus minimus at hip internal rotation in individuals with FADIR-positive pain, using ultrasound and MR imaging analysis. Our hypothesis was that, at the hip internal rotation position in which the gluteus minimus slacks, the gluteus minimus and joint capsule are oriented in the same direction to a greater extent in the FADIR-positive group than in the control group due to specific problems, such as a lack of smooth movement between the gluteus minimus and joint capsule.

## Methods

### Participants

Fifty-nine healthy volunteers (39 men and 20 women; mean age, 20.7 ± 2.6 years) at Morinomiya University of Medical Sciences were investigated. The study design was approved by the ethics committee of Morinomiya University of Medical Sciences (#2022-013), and all procedures were performed in accordance with the Declaration of Helsinki (last modified in 2013) and the Japanese guidelines entitled “Ethical Guidelines for Medical and Health Research Involving Human Subjects.” All participants provided written informed consent.

A flow diagram of the study participant enrollment is summarized in Additional File 1. Participants with no history of hip surgery were included. Participants underwent a physical examination in the supine position, such as passive hip range of motion and the FADIR test. Passive range of motion was evaluated, especially hip internal/external rotation with the hip flexed to 90°, to confirm the absence of an apparent range of motion limitation. Reduced internal rotation was defined as ≤ 10° internal rotation with the hip flexed to 90° [7, 18–20], and two participants were excluded. The FADIR test was performed on the remaining 57 participants to assess the presence of hip pain. Twelve participants (14 hips) were identified as having hip pain in the FADIR test and 45 participants (90 hips) were identified as without hip pain. Of the 12 participants with 14 hips, 4 participants with 4 hips had other than anterior hip pain; therefore, the remaining 8 participants with 10 hips (5 men and 3 women; mean age, 21.5 ± 4.5 years) were included in this study as a FADIR-positive group. In addition, 10 age-matched participants with 10 hips (7 men and 3 women; mean age, 21.8 ± 3.8 years) were selected as a control group from 45 participants without hip pain in the FADIR test. The characteristics of both groups are shown in Table 1.

**Table 1:** Participant characteristics

	FADIR positive (10 hips)	Control (10 hips)	*P* value
Age (years)	21.5 ± 4.5	21.8 ± 3.8	0.88
Height (m)	1.66 ± 0.09	1.68 ± 0.08	0.53
Weight (kg)	55.3 ± 9.2	59.0 ± 6.9	0.35
BMI (kg/m^2^)	19.8 ± 1.5	20.7 ± 1.5	0.27
Hip range of motion (°)			
Internal rotation	23.5 ± 5.0	30.5 ± 4.2	0.0047
External rotation	36.0 ± 8.0	39.0 ± 6.6	0.40
Total rotation	59.5 ± 11.1	69.5 ± 9.1	0.050

Data are expressed as mean ± standard deviation

BMI = body mass index, FADIR = flexion adduction internal rotation

### Ultrasound imaging analysis

Ultrasound assessment of the hip was performed using an ultrasound scanner (Aplio 300; Canon Medical Systems, Tokyo, Japan) equipped with a 14-MHz linear transducer (PLT-1005BT; Toshiba, Tokyo, Japan). Long-axis B-mode images of the hip in the coronal plane posterior to the anterior inferior iliac spine were acquired (960 × 720 pixels, 8 pixels/mm), and the gluteus minimus and joint capsule were visualized in the sitting position fixed by an isokinetic dynamometer (Biodex System 4; Biodex Medical System Inc., Shirley, NY, USA). For the sitting position, the trunk and pelvis were set at a neutral position, the hip was flexed at 90° with neutral abduction/adduction, and its rotation angle (external/internal rotation) was set to the following four conditions: 20° and 10° external rotations, 0° external/internal rotation, and 10° internal rotation.

**Fig. 1:**
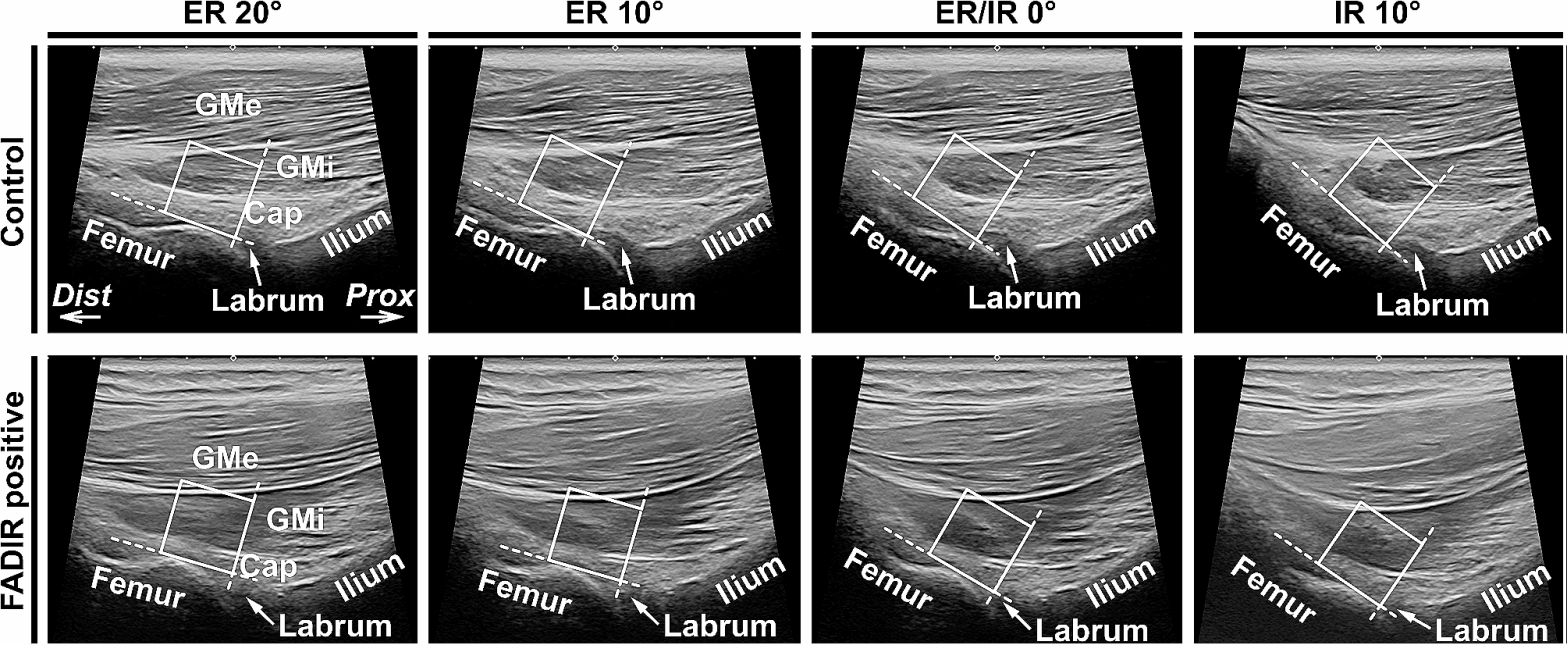
Ultrasound images of the gluteus minimus and joint capsule under rotation in hip flexion. Long axis images of the hip in the coronal plane posterior to the anterior inferior iliac spine. The upper row indicates images of the control group and the lower row, flexion adduction internal rotation (FADIR)-positive group. Images at 20° and 10° external rotations (ER 20° and 10°), 0° external/internal rotation (ER/IR 0°), and 10° internal rotation (IR 10°) in 90° hip flexion are shown from left to right. Squares indicate the regions of interest for orientation measurement

**Fig. 2:**
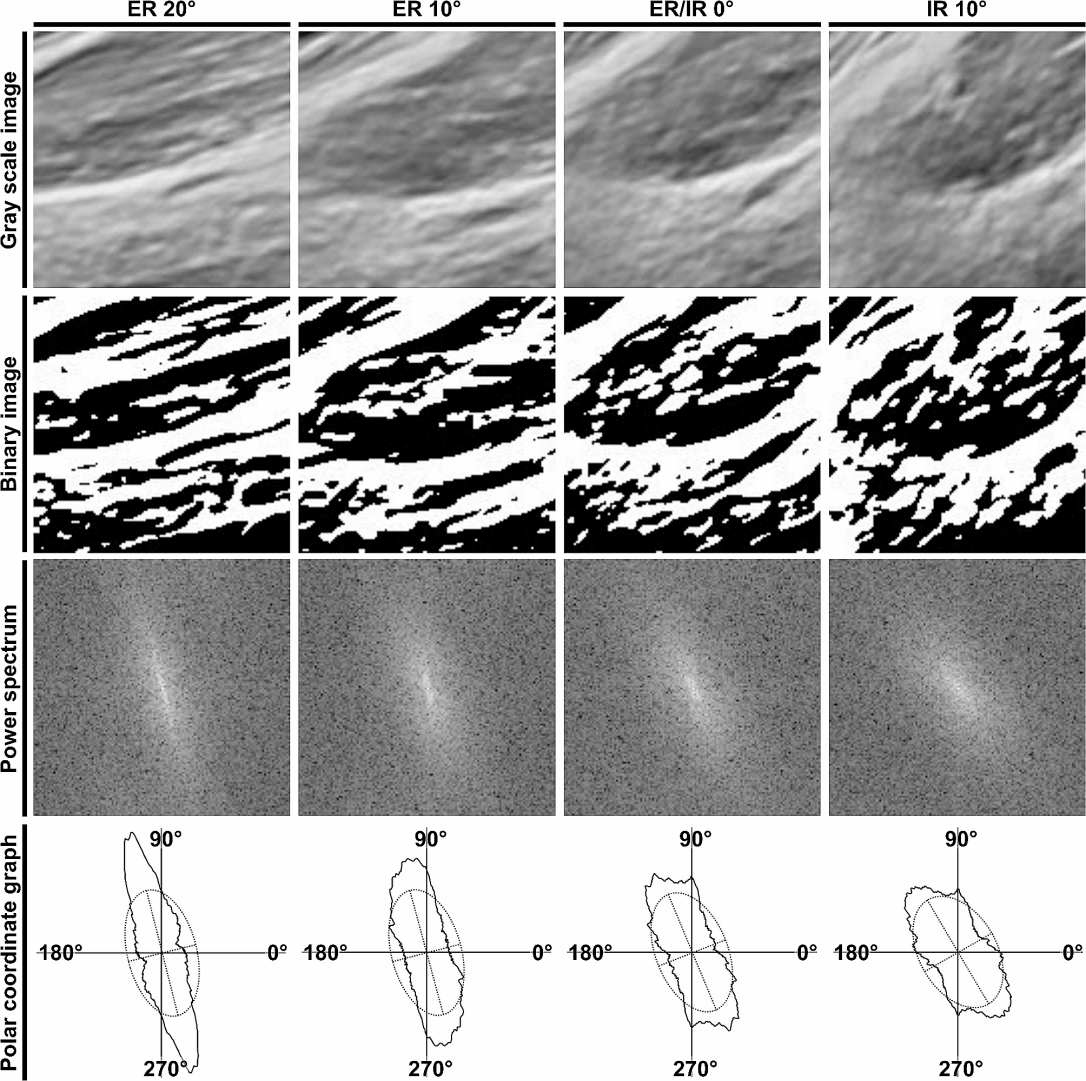
Orientation measurements of the gluteus minimus and joint capsule in the control group. Analysis of ultrasound images in the control group. The following are shown in order from the top row: the grayscale image of the region of interest shown in Fig. 1 (upper row), the corresponding binary image, the power spectrum picture after two-dimensional Fourier transform of the binary image, and the orientation distribution denoted by the angle and mean amplitude (polar coordinate graph) and its elliptic approximation. The ratio of the major and minor axes of the ellipse can be regarded as the orientation intensity. Images and their analysis process at 20° and 10° external rotations (ER 20° and 10°), 0° external/internal rotation (ER/IR 0°), and 10° internal rotation (IR 10°) in 90° hip flexion, in order from left to right, are shown

**Fig. 3:**
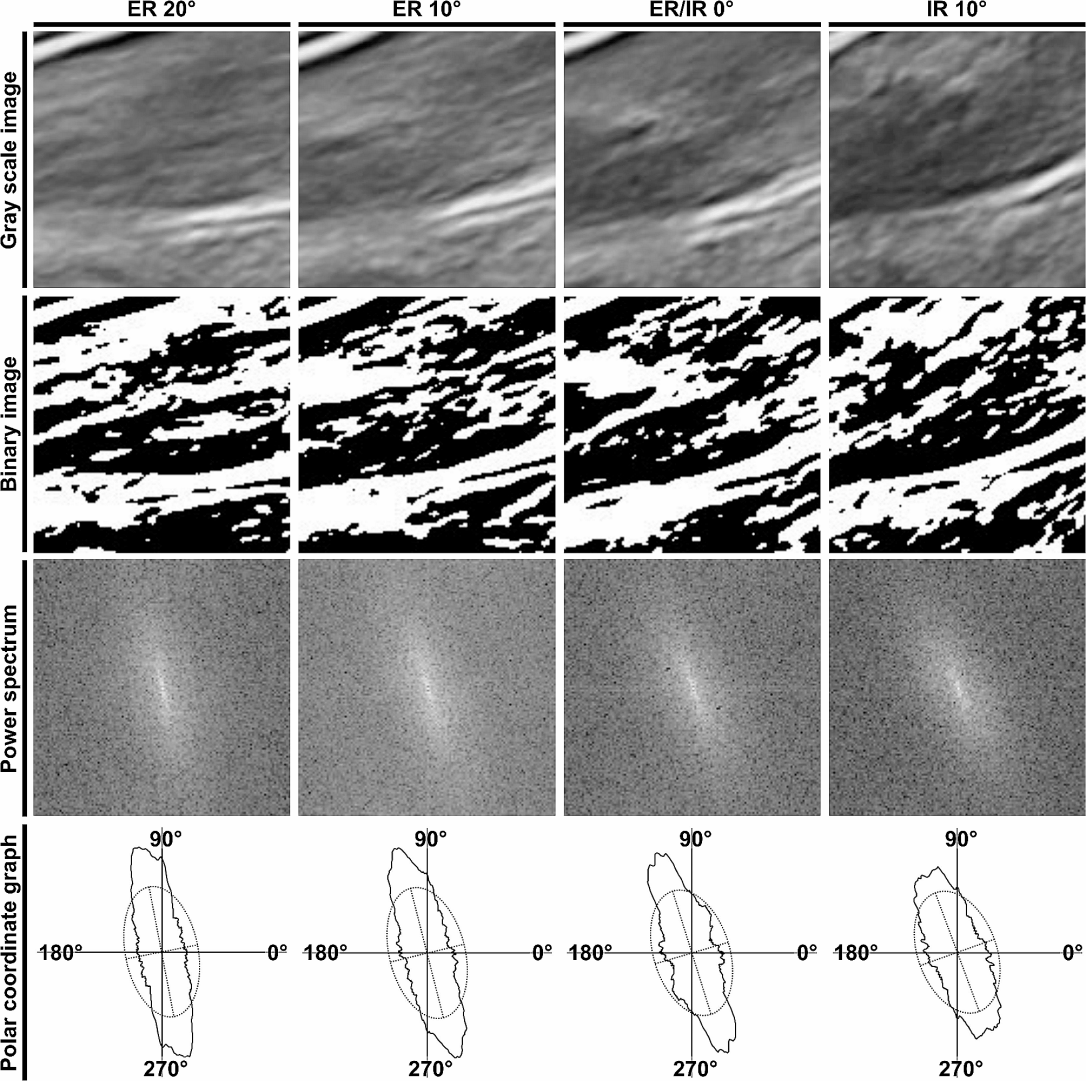
Orientation measurements of the gluteus minimus and joint capsule in the FADIR-positive group. Analysis process of ultrasound images in the FADIR-positive group. The following are shown in order from the top row: the grayscale image of the region of interest shown in Fig. 1 (upper row), the corresponding binary image, the power spectrum picture after two-dimensional Fourier transform of the binary image, and the orientation distribution denoted by the angle and mean amplitude (polar coordinate graph) and its elliptic approximation. The ratio of the major and minor axes of the ellipse can be regarded as the orientation intensity. Images and their analysis process at 20° and 10° external rotations (ER 20° and 10°), 0° external/internal rotation (ER/IR 0°), and 10° internal rotation (IR 10°) in 90° hip flexion, in order from left to right, are shown. FADIR = flexion adduction internal rotation

Based on the recorded ultrasound images at the four hip rotation conditions, orientation measurements of the gluteus minimus and joint capsule were performed and quantitatively compared between the FADIR-positive and control groups. The region of interest in the image was set parallel to the axis of the neck of the femur and in contact with the head of the femur and acetabular labrum (Fig. 1) and cropped as an 8-bit grayscale image of 128 × 128 pixels using ImageJ software version 1.53 (National Institutes of Health, Bethesda, MD, USA) (Figs. 2 and 3; top row). Thus, the region of interest was set to include the muscular belly portion of the gluteus minimus proximal to the area where the gluteus minimus transitions to its tendon. This cropped image was then analyzed by the two-dimensional discrete Fourier transform using fiber orientation analysis software (FiberOri8single03; Tsukuba University, Ibaraki, Japan) [21]. The analysis process using this software is summarized as follows. First, the image was binarized (Figs. 2 and 3; second row from top). Second, the power spectrum was obtained by the two-dimensional discrete Fourier transform based on the binary image (Figs. 2 and 3; third row). The power spectrum was then divided radially into 180 regions from a central angle of 0°–180°, and the mean amplitude was obtained for each region. Finally, the orientation distribution was denoted by the angle and mean amplitude as a polar coordinate graph, and an elliptic approximation was created (Figs. 2 and 3; bottom row). For each angular component, larger amplitudes indicate a greater number of fibers at that angle. Therefore, if the fibers are not preferentially arrayed, all angular components have similar amplitudes, and the approximated ellipse approaches a circle. Conversely, if the fibers are preferentially arrayed, there are many fibers with a particular angular component, which accordingly has a large amplitude; therefore, the major axis of the approximated ellipse is longer than the minor axis. Consequently, the ratio of the major and minor axes of this ellipse can be regarded as the orientation intensity (≥ 1). The orientation intensity at the region of interest in the FADIR-positive and control groups were compared under four conditions: 20° and 10° external rotations, 0° external/internal rotation, and 10° internal rotation. A large orientation intensity means that the fibers in the region of interest are oriented in the same direction [21–23]. To assess reliability, visualization of the hip long-axis image and measurement of the orientation intensity were performed twice by a single examiner, and the average of two measurements was recorded for statistical analysis. Intraclass correlation coefficients were calculated to determine the intrarater reliability of each measured value.

### MR imaging analysis

After ultrasound imaging analysis, 3 hips of 3 participants were randomly selected from each of the control (3 men; mean age, 24.7 ± 5.9 years) and FADIR positive groups (2 men and one woman; mean age, 20.3 ± 0.5 years) for MR imaging analysis. Imaging of the gluteus minimus and joint capsule in the coronal plane was performed using a 0.3 T MR imaging system (AIRIS Vento; FUJIFILM Healthcare Systems Corp., Tokyo, Japan) with a joint coil (MR-JCR-103; FUJIFILM Healthcare Systems Corp., Tokyo, Japan). Coronal hip images were acquired as T_1_-weighted images using a spin-echo sequence. The MR parameters were as follows: repetition time/echo time, 400/25.0 ms; flip angle, 90°; 260 × 224 matrix; recon matrix 512; 25-cm field of view; 5.0-mm sections; 1.0-mm interval; and acquisition time, 4 min 30 s per position. The participants were placed in a semi-prone position with the examination hip on top, and each hip was maintained at the center of a magnet bore. The trunk and pelvis were set at a neutral position, the hip was flexed at 90° with neutral abduction/adduction, and its rotation angle (external/internal rotation) was set to approximately 10° internal rotation (the lower leg was placed on a fixed device with a defined angle, Shamen 10; Takada bed, Ltd., Sennan, Japan). To confirm whether the internal rotation angle was sufficient, MR images were also taken with 0° external/internal rotation positions in hip 90° flexion (the lower leg was placed on an examination bed without the fixed device). Ten slices were imaged from the superior end of the greater trochanter, including the anterior inferior iliac spine. Using ImageJ software, the axis angle of the neck of the femur confirmed that the MR images were acquired at a hip internal rotation of 9.2 ± 1.6°. The acquired MR images were compared between the FADIR-positive and control groups to verify the results of the ultrasound imaging analysis.

### Statistical analyses

Statistical tests were performed using SPSS (version 27.0; IBM Corp, Armonk, NY, USA). Distributions of all measurements consistently passed the Shapiro-Wilk test, and the data are expressed as the mean and standard deviation. Using the unpaired t-test, participant characteristics in the FADIR-positive and control groups were compared. We then performed a two-way repeated measures analysis of variance (ANOVA) on the orientation intensity of the ultrasound image visualizing the gluteus minimus and joint capsule, with FADIR positive/control groups as the between-groups factor and the four hip rotation conditions as the within-group factor. If there was a significant difference in the between-groups factor, a statistical comparison of the FADIR-positive and control groups for each rotation angle was performed using the unpaired t-test. Significance levels of the two-way repeated measures ANOVA and unpaired t-test were set at *p* < 0.05, and the effect size was also calculated for each statistical analysis. In addition, a post-hoc power analysis was performed using G*Power software (version 3.1.9.6); the power of the between-groups factor was 0.98.

The intraclass correlation coefficients of the orientation intensity were also determined. The qualitative cut-offs for intraclass correlation coefficient values were reported as follows: poor, < 0.40; fair, 0.40–0.59; good, 0.60–0.74; and excellent, 0.75–1.0 [24]. The intraclass correlation coefficients of the orientation intensity at 20° and 10° external rotation, 0° external/internal rotation, and 10° internal rotation were 0.85 [95% CI 0.63–0.94], 0.90 [95% CI 0.74–0.96], 0.87 [95% CI 0.67–0.95], and 0.92 [95% CI 0.80–0.97], respectively. All ICCs were ≥ 0.63 (range, 0.63–0.97), indicating good or excellent agreement.

## Results

### Participant characteristics

The hip range of motion of internal rotation with the hip flexed to 90° in the FADIR-positive group was significantly less than that in the control group (*p* = 0.0047), although there were no significant differences in the other characteristics (Table 1).

### Ultrasound imaging and its orientation intensity analysis

**Fig. 4:**
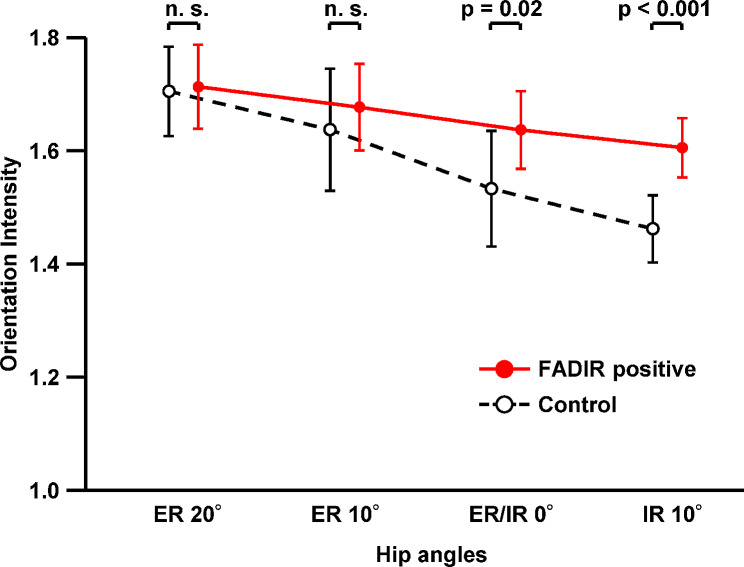
Orientation intensity comparison of the gluteus minimus and joint capsule in control and FADIR-positive groups. The orientation intensity in the FADIR-positive group was significantly higher than that in the control group at 0° external/internal rotation (ER/IR 0°) and 10° internal rotation (IR 10°) in 90° hip flexion (two-way repeated measures analysis of variance, main effect for group, *p* < 0.001; post-hoc unpaired t-test, *p* = 0.02 at ER/IR 0° and *p* < 0.001 at IR 10°). FADIR = flexion adduction internal rotation

Based on the ultrasound imaging analysis in the coronal plane, the gluteus minimus and joint capsule at the 20° or 10° external rotation position in the hip at 90° flexion showed fibers oriented in the same direction regardless of group (FADIR positive/control groups) (Fig. 1). In contrast, although the 10° internal rotation position also showed fibers oriented in the same direction in the FADIR-positive group, fibers were oriented in a different direction in the control group. As a result of the fiber orientation measurement (Figs. 2 and 3), two-way repeated measures ANOVA showed significant main effects between groups (FADIR positive/control groups; F = 15.47, *p* < 0.001, effect size η^2^ = 0.11) and within-group (four hip rotation conditions; F = 17.32, *p* < 0.001, η^2^ = 0.35) factors, but no significant interaction effect (F = 2.61, *p* = 0.058, η^2^ = 0.053) (Fig. 4). At the 20° and 10° external rotation positions, there were no significant differences in orientation intensity between FADIR-positive and control groups (FADIR-positive vs. control at 20° external rotation, 1.71 ± 0.07 vs. 1.70 ± 0.08, *p* = 0.80, effect size *r* = 0.06; at 10° external rotation, 1.67 ± 0.08 vs. 1.64 ± 0.11, *p* = 0.38, *r* = 0.21). In contrast, orientation intensities at 0° external/internal rotation and 10° internal rotation in the FADIR-positive group were significantly larger than those in the control group (FADIR-positive vs. control at 0° external/internal rotation, 1.63 ± 0.07 vs. 1.53 ± 0.10, *p* = 0.022, *r* = 0.51; at 10° internal rotation, 1.60 ± 0.05 vs. 1.46 ± 0.06, *p* < 0.001, *r* = 0.79). Therefore, for the gluteus minimus and joint capsule at 0° external/internal rotation and 10° internal rotation in the hip at 90° flexion, the fibers in the FADIR-positive group were significantly more oriented in the same direction than were those in the control group.

### MR imaging analysis

**Fig. 5:**
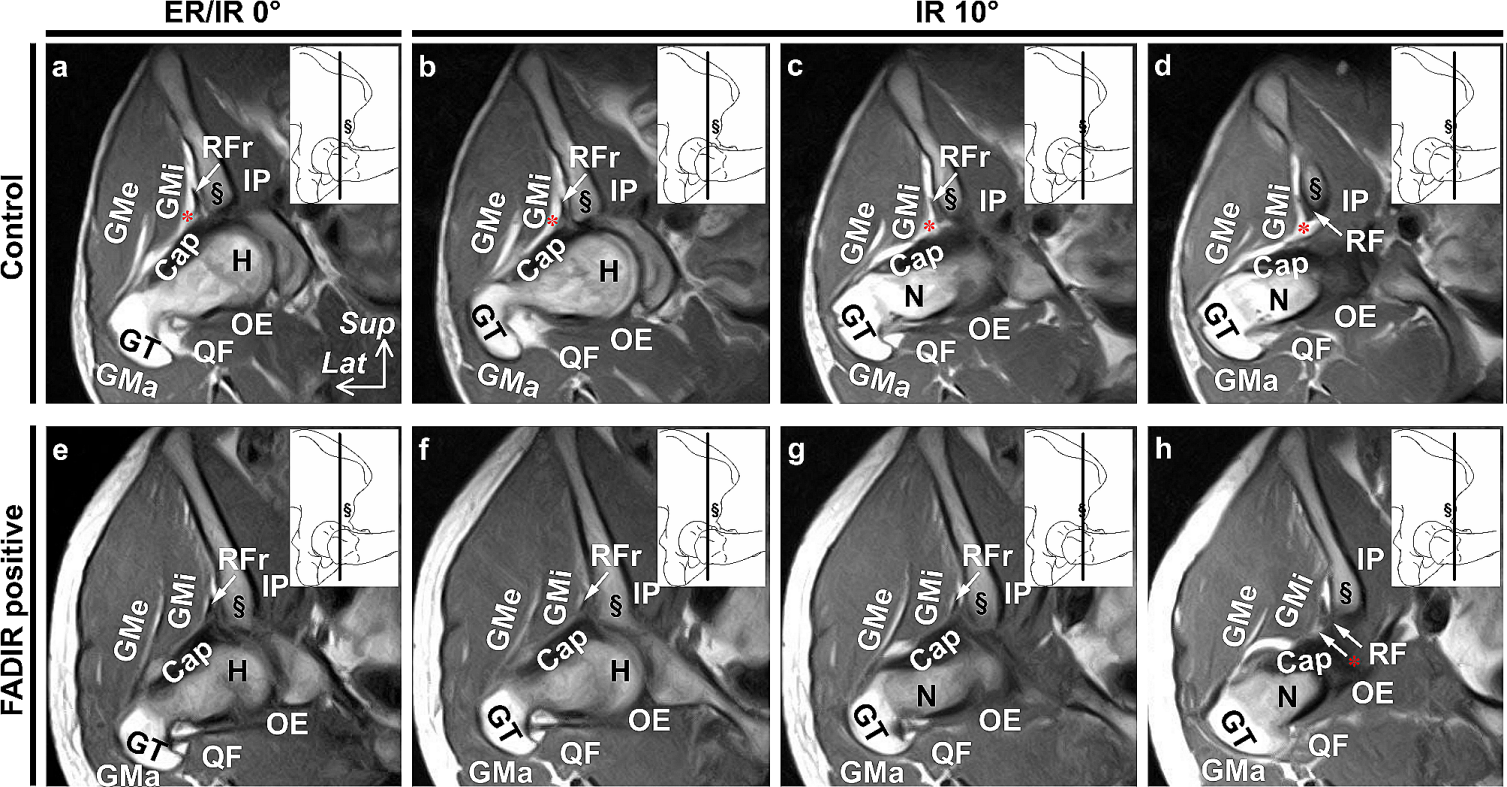
MR images of the gluteus minimus and joint capsule under internal rotation in hip flexion. Coronal section images of the hip in the control (**a**-**d**) and flexion adduction internal rotation (FADIR)-positive groups (**e**-**h**). The locations of each coronal section are indicated by black lines in each upper right panel, which is a schematic illustration of the lateral aspect of the right hip (90° flexion). Images were acquired at 0° external/internal rotation (ER/IR 0°; **a** and **e**) and 10° internal rotation (IR 10°; **b-d** and **f-h**) in 90° hip flexion. Asterisks (*) indicate the proximal spaces between the gluteus minimus (GMi) and joint capsule (Cap), which are prominent in the control group. GMa = gluteus maximus, GMe = gluteus medius, GT = greater trochanter, H = head of femur, Ip = iliopsoas, MR = magnetic resonance, N = neck of femur, OE = obturator externus, QF = quadratus femoris, RF = rectus femoris, RFr = reflected head of the RF, Section mark (§) = anterior inferior iliac spine, *Lat* = lateral and *Sup* = superior

MR images of the gluteus minimus and joint capsule at internal rotation positions in hip flexion were compared between the FADIR-positive and control groups. At the coronal slice level from the anterior to posterior edges of the anterior inferior iliac spine, the proximal fatty tissue or loose connective tissue spaces between the gluteus minimus and joint capsule were prominent in all individuals of the control group (Fig. 5a-d). However, in all individuals of the FADIR-positive group, such a proximal space was not apparent, and the gluteus minimus and joint capsule were in close contact with each other (Fig. 5e-h).

## Discussion

The present study revealed that at hip internal rotation at 90° flexion, ultrasound images of the gluteus minimus and joint capsule showed that fibers in the FADIR-positive group were significantly more oriented in the same direction than those in the control group. Moreover, based on the MR images, the proximal loose connective tissue spaces between the gluteus minimus and joint capsule were prominent at hip internal rotation in the control group, although such proximal space was not apparent in the FADIR-positive group.

Several *in-vivo* imaging studies previously suggested that in individuals with FADIR-positive pain, the osteological relationship between the acetabulum and femur at the hip internal rotation position in flexion or the FADIR position itself has been visualized as different from those of healthy people [25–27]. However, in individuals with FADIR-positive pain, there has been little focus on how the characteristics of the anterior hip soft tissue at the hip internal rotation position in flexion or FADIR position are different from those of healthy people. Relevant to the anterior hip soft tissue findings in the present study regarding the gluteus minimus and joint capsule, it is anatomically well known that the gluteus minimus tendon distally connects to the joint capsule [28–31]. In contrast, the loose connective tissue is proximally distributed between the gluteus minimus and joint capsule [16, 29], and such loose connective tissue generally promotes movement between structures [32, 33]. The present study verified that proximal loose connective tissue spaces between the gluteus minimus and joint capsule were prominent during hip internal rotation in the MR images of the control group. In the FADIR-positive group, this space was not sufficient, and therefore, the ultrasound imaging analysis can be interpreted as the movement between the gluteus minimus and joint capsules not being smooth, resulting in the same fiber direction.

Our study findings suggest that the lack of smooth movement between the gluteus minimus and joint capsule due to a pathological problem in the loose connective tissue between them may be related to anterior hip pain and contribute to limited hip internal rotation in FADIR-positive individuals. Pathological changes in loose connective tissue have recently received attention because advanced interventions for these pathologies (hip arthroscopy or ultrasound-guided injection) were effective in reducing anterior hip pain [14, 15]. Therefore, the possibility that these pathological changes in the loose connective tissue might also be one of the pathological causes of anterior hip pain in the FADIR test, other than a bony deformity or acetabular labrum, is important for a better understanding of hip pain pathogenesis.

Our study has several limitations. First, we cannot clarify whether the interrelationships between the gluteus minimus and joint capsule directly affect anterior hip pain in the FADIR test or limited hip internal rotation in FADIR-positive individuals without an interventional study, and our pathological theory was speculative. Future studies should evaluate whether interventions for the gluteus minimus, joint capsule, and loose connective tissue between them reduces anterior hip pain in the FADIR test or improves limited hip internal rotation in FADIR-positive individuals. Second, we did not perform a radiographic evaluation to avoid radiation exposure; thus, individuals with some bony deformities could not be excluded. Finally, the sample size was relatively small. However, sufficient effect size and power were obtained, and we also compensated for the small sample size with multidimensional analysis, including ultrasound and MR imaging analysis.

In conclusion, at hip internal rotation in 90° flexion, the muscular belly portion of the gluteus minimus and joint capsule were oriented in the same direction to a greater extent in the FADIR-positive group than in the control group owing to a morphological change in the loose connective tissue between them. The pathological changes in the loose connective tissue may inhibit smooth movement of the gluteus minimus relative to the joint capsule in individuals with FADIR-positive pain.

### Electronic supplementary material

Below is the link to the electronic supplementary material.

Additional File 1. Flow diagram of the study participant enrollment

## Data Availability

The datasets used and/or analyzed during the current study are available from the corresponding author upon reasonable request.
