# Supplementary material for: In vivo interrelationships between the gluteus minimus and hip joint capsule in the hip internal rotation position with flexion
Source: BMC Musculoskelet Disord. 2024 Jan 23;25:87. doi: 10.1186/s12891-024-07188-5 (PMC10804474; doi:10.1186/s12891-024-07188-5)
Supplement: Supplementary file 1 — Additional File 1. Flow diagram of the study participant enrollment [file 12891_2024_7188_MOESM1_ESM.pdf]

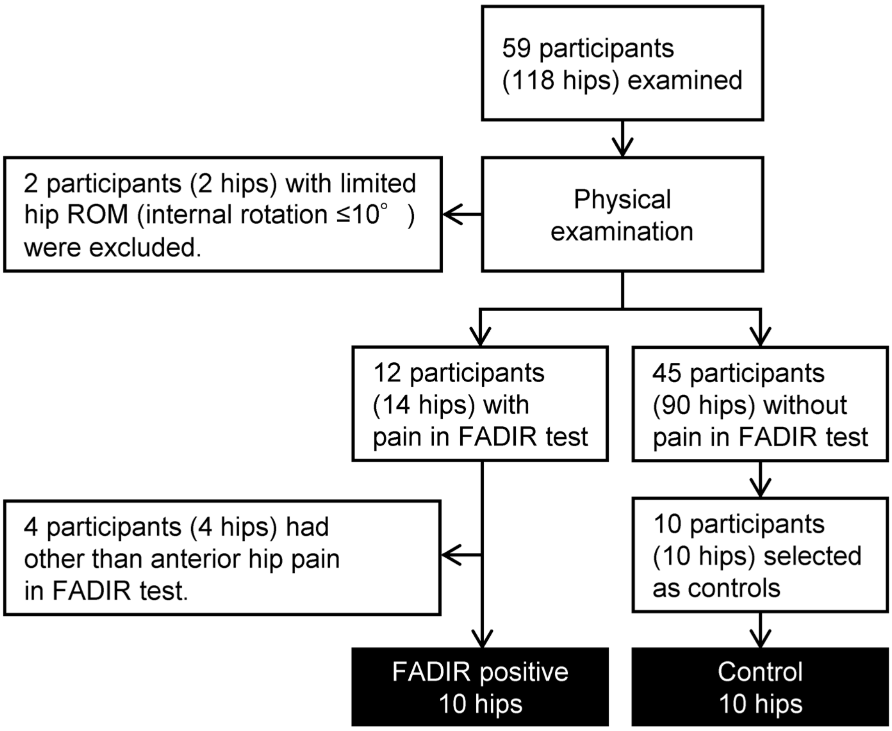

Additional File 1. Flow diagram of the study participant enrollment.

FADIR = flexion adduction internal rotation, ROM = range of motion
